# Ethical Issues of Transplanting Organs from Transgenic Animals into Human Beings

**Published:** 2014-10-04

**Authors:** Shima Behnam Manesh, Reza Omani Samani, Shayan Behnam Manesh

**Affiliations:** 1 Department of Epidemiology and Reproductive Health at Reproductive Epidemiology Research Center, Royan Institute for Reproductive Biomedicine, ACECR, Tehran, Iran; 2 Medical Ethics and History of Medicine Research Center, Tehran University of Medical Science, Tehran, Iran

**Keywords:** Transgenic Animals, Organ Transplantation, Animal Welfare, Xenotransplantation

## Abstract

One of the most important applications of transgenic animals for medical purposes is to transplant their organs into human’s body, an issue which has caused a lot of ethical and scientific discussions. we can divide the ethical arguments to two comprehensive groups; the first group which is known as deontological critiques (related to the action itself regardless of any results pointing the human or animal) and the second group, called the consequentialist critiques (which are directly pointing the consequences of the action). The latter arguments also can be divided to two subgroups. In the first one which named anthropocentrism, just humankind has inherent value in the moral society, and it studies the problem just from a human-based point of view while in second named, biocentrism all the living organism have this value and it deals specially with the problem from the animal-based viewpoint. In this descriptive-analytic study, ethical issues were retrieved from books, papers, international guidelines, thesis, declarations and instructions, and even some weekly journals using keywords related to transgenic animals, organ, and transplantation. According to the precautionary principle with the strong legal and ethical background, due to lack of accepted scientific certainties about the safety of the procedure, in this phase, transplanting animal’s organs into human beings have the potential harm and danger for both human and animals, and application of this procedure is unethical until the safety to human will be proven.

## Introduction

Today, organ transplantation has attracted many attentions, and has socially caused a lot of worry. The main problem is the imbalance between the organ transplant request and the number of organs ready to transplanting. At present, patients tolerate not only the pain caused by organ malfunction, but also psychological tensions while waiting to receive healthy organs. In 2008, in America, it was estimated that almost 97000 people were in the waiting list to receive organs while every day, 13 people died due to lack of required healthy organs at a crucial moment (1).

Nowadays, there is remarkable progress in different organ transplant techniques which are classified into two general types. Homograft is used when both the donor and the recipient of the cell have tissue or the organ belongs to one biological species, despite their genetic differences. Another method, called Xenograft, is applied when the donor and the recipient belong to the two different biological species (2). Xeno means stranger and alien in Latin (3). Therefore, based on the definition of this technique, each process involving transplant, implantation of animal cells, tissue or the organs injection to a human receiver is called Xenograft (4).

This method based on the degree of evolutionary and biological closeness between the donor and the recipient is classified in two types, entitled consonant and disconsonant (4).

In the consonant type, two biological species are close, both genetically and biologically for example transplantation from various monkey species to humans, while in disconsonant there is not an obvious genetic closeness, like transplanting from pigs to humans (5).

Historical studies have indicated that with developing skills and knowledge in the field of medicine, human has increased his exploitations of animals in order to transplanting their tissues and organs into human beings. In 1628, animal’s blood was flow in human veins (6). Following that in 1682, a Russian physician tried to save a man through transplanting a part of dog’s skull. Catgut suture, originally made from intestines of sheep, is well known among physicians. Nonetheless, attempts to use an organ completely started in early 20^th^ century. In 1963, Chimpanzee’s kidney was transplanted into 13 patients, and in 1984, baboon’s heart was transplanted into a baby with heart failure; the child died 20 days after the operation. In 1992, baboon’s liver was transplanted; the patient died 70 days later (7). From a historical perspective, various examples of transplanting animal’s organ into human can be reported, but the common fact is that lifespan of the recipient after the operation was very short and that all patients died in various stages of acceptance (8).

A major problem in organ transplant from animal to human is recipients’ immune response. The transplanted organ is identified as alien. Therefore, the immune system rejects it to protect the body. Based on time, this immunological reaction occurs in three forms of hyper-acute, acute, and chronic rejections. In the first form, the transplanted organ is rejected within only seconds or minutes after the transplantation; in the second form, this rejection happens after some days to a week; and in the third, in longer-term, within some weeks to years (9).

In xenograft, mostly, we are dealing with hyper acute and acute rejections (10). In order to overcome this obstacle, scientists and physicians have shown great interest in using transgenic animals through applying genetic engineering. So, it is expected that these animals whose genomes have been modified or manipulated are going to be wildly used in medical or agricultural fields (11).

*Escherichia coli (E. coli)* was the first bacterium to be genetically changed. Since then, this technology has improved a lot and is being conducted on plants and animals. The first genetically engineered mouse was created almost three decades ago, and up to now, many transgenic farm animals have been produced (12). Currently, transgenic animals are widely used in biology, medicine, and biotechnology. It is expected that transgenic animals can provide an applicable source for human organ transplantations.

Nevertheless, regarding organ transplant, pigs are now focused. Although primates can be selected as good options due to genetic closeness, many of them, like various monkey species, are in danger of extinction. While their pregnancy and growth rate are also long, so they are not widely studied. However, the pig is a domestic animal whose keeping and breeding is easy, growing is rapid, time of pregnancy is short, and organ size is close to the human. So it is chosen as the first option (13). Currently, human proteins have been produced in pigs’ internal organs using genetic engineering techniques. In this study, we tried to present and organize all the ethical arguments in a relatively new way and discuss and present answers and finally give the ethical justification of the subject according to current conditions.

In this descriptive-analytic study, ethical issues were retrieved from books, papers, international guidelines, thesis, declarations and instructions, and even some weekly journals using keywords related to transgenic animals, the organ, and transplantation. Then arguments were organized in a relatively new manner. Each argument is discussed and possible answers are presented. Then author’s scheme of the ethical justification and its conditions is presented as the conclusion.

### Ethical issues

Ethical arguments expressed about transplantation from transgenic animals to human are wide and have various ranges, based on their subject and nature. They are given below in different classifications in order to help make the clear discussion. We presented the arguments in two main categories: "deontological" and "consequentialist" (14). In the first group, ethicists believe that the very nature of such an action is unethical and unacceptable regardless of the consequences of procedure and its effects on human or animals. In the second group, the transplant process is regarded as unacceptable because the contradictory outcomes occur for both humans and animals (15).

### Deontological arguments

The most important criticism expressed is the playing God and being unnatural. In the first group which partly possesses theological characteristics, critics believe that mankind has become so revolutionary and rebellious trying to take the place of God. In addition, using genetic engineering, mankind is able to create creatures that have never existed in the nature. Therefore, he is passing from being a creation to being a creator, more th an ever (14).

Second argument says that basically, transgenic animal’s organ transplantation into human is "unnatural" and is considered as an inappropriate interfere in nature, both in order and structure that are intrinsically good.

The most important part of this argument is modifying or manipulating animal genomes, whereas breaking usual boundaries among biological species caused by human interference is something which never happens naturally (16).

To answer this argument, it must be said that the exact meaning of being natural isn’t completely clear, so being natural is used mostly as opposite to "being artificial" or "man-made", but it can be used as eternal, usual, favored, appropriate, by itself, intrinsic, etc. Furthermore, there is no reason for accepting that "whatever is natural is necessarily good and what is unnatural is bad and inappropriate". For example, no logical mind can accept that because flood is a "natural" phenomenon, then it is "good", while controlling flow of water through constructing dams is "bad" and inappropriate since it’s a "man-made"(17). In addition, some philosophers like Bernard Rollin have mentioned that the result of accepting such an argument is a complete stop of life. He has added that mankind has done everything in history to survive, like all inventions, constructing dams over rivers, fighting diseases, and using methods to control pregnancy, are assumed to be unnatural events (18).

These criticisms were too strong to be considered due to their inability in providing proper answers to the opponents not only regarding transgenic animals’ organ transplantation into human, but also in all dimensions of genetic engineering and stem cell researches.

### Consequentialist arguments

These arguments consider the process of transgenic animals’ organ transplantation into human to be unacceptable due to their consequences and complication. According to those who are influenced by the complications, the nature of the arguments and the ethical schools are different.

### Anthropocentric ethics

This school has always been a dominant vision in the history of ethics. Only mankind is acknowledged as a member of ethical society, and based on some religious considerations or some outstanding characteristics like intelligence, soul, speech, and sophisticated communications, mankind has always been in the center of attention and summit of ethics pyramid, while other creatures have been considered inferior to human (19). As a result, philosophers that are in favor of this viewpoint have examined unpleasant effects of xenotransplantation from transgenic animals’ only for "human". From this point of view, possible effects that can happen to "human beings" can be classified into two groups of "individual" and "collective".

### Individual human-based (anthropocentric) criticism

In an individual dimension, effects are considered for a "person" receiving the animal organ. One of the most important issues is the lack of certainty and proper scientific evidence regarding transfer of viruses and diseases from animals to the recipient. As a result of living with animals or using animal products, mankind has been infected with some animal diseases like cow madness disease or chicken influenza.

It is expected that about animals’ organ transplantation into human' body, the possibility of viruses and micro-organisms transmitted increases, as well. Considering this possibility that can easily endanger patient’s welfare, well-being, and even life, unfortunately there are many uncertainties which doctors have not yet been able to solve. It has been proved that micro-organisms are living in the body of a creature without any bad effects (called: natural microflora). Micro-flora of species can be harmful and even lethal for the others. On the other hand, functions of viruses are not totally predictable and they can change in nature due to some jumping reaction; therefore, it may have irreversible effects in the new host. For instance, a type of herpes simplex virus (HSV) lives as natural flora and harmless in spider monkey’s body, but when it transfers into other species body, it causes lymphoma or other types of blood cancers (8).

Besides, some microorganisms living in animals are yet "unknown", so mankind has less knowledge of their nature and effects.

In order to increase the success of transplantation, the organ receiver should take great amounts of immunosuppressive drugs before and after the procedure to prevent organ rejection, but immunosuppressive drugs will also intensify the possibility of infectious diseases. This may change the immune response to the viruses which may have no effect on an immune competent person. Besides, there are so many factors affecting the immune system, like stress caused by financial, nutritional, and physical problems, so patient’s immune system could not have the usual function. In this situation, the possibility that the recipient catches infectious diseases is more than normal people (20).

In addition, the incubation period of some viruses and infectious diseases is long, so they will appear some years after the operation. Therefore, it is not clear how long the patient have to suffer the side effects of transplantation. It is obvious that in transgenic animals’ organ transplantation, human knowledge faces a world of uncertainty. According to the precautionary principle, this weakness makes the ethical justification of this transplantation method almost impossible (21).

The precautionary principle is defined as the principle for making practical decisions when the condition has scientific uncertainty. This principle, supported by ethics, is defined by Gardiner, and has three elements: threat of harm, uncertainty of impact or causality, and the precautionary response. So, the researchers should identify all possible harms and dangers before implementing a new technique, whereas this procedure with such a level of certainty seems to be not ethically justified (22).

Furthermore, due to the mentioned risks, transplant to the recipient is a new beginning and difficult period, because organ transplant to the recipient should be under close follow-up for an indefinite period of time. Also, this can affect the most private aspects of human’s life, like nutrition and sexual relationships. Moreover, regarding infections, they have to live in a completely quarantined place and isolated from others. This situation is obviously against the most basic and essential human right i.e. freedom and establishment of relationships with the others.

Considering all above, the necessity of getting "informed consent" from the organ recipient is also obvious. It is clear that the nature of informed consent philosophy is the permission of patient about the procedure after receiving enough information about treatment and side effects, and in return, the patients will be safe from types of treatment that are unwanted or that are incompatible with their beliefs (21).

Considering transgenic animals’ organ transplantation, the meaning of informed consent changes to some extent because the person should express his/her consent to be under restriction and care after the operation in which timing is uncertain. Therefore, the range of consent is remarkably transferred to after the operation and patients express their informed consent knowing that they may not have the normal life after the operation. In addition, the nature of consent changes from voluntarily to obligatory. Unlike other treatment processes in which the patient has the right to return the consent, here, patients’ withdrawal, especially in the case of appearance of infectious diseases, is not possible at all. The patient must not only accept the operation but also be obligated for cooperation during follow-up treatment to control side effects. Therefore, it is impossible to withdraw from informed consent in phases after transplant process (23).

Another important criticism deals with this ambiguity, and lack of scientific certainty is whether the transplanted organ can function as desired in the human body or not. It has been mentioned that some organs and especially liver have various and complicated functions that may be different in various biological species and that if transferred, can’t have the expected function in human body (24, 25).

Besides, the position of organ in animal’s body is an important issue. A major concern is that pig’s heart and lung, which are positioned horizontally in body, can function in human body in vertical form or not? This difference may interfere in pulmonary circulation between heart and lung, and this will result in the death of patient.

The last criticism of individual dimension set is about the fact that transgenic animal’s organ transplantation into human can cause major psychological and personality problems for the recipient. It has been proved that the individual has certain idea about physical shape of his/her body which forms his/her character. Therefore, acceptance of animal’s organ transplantation into human seems to have some controversies for both the patient and society (25).

Regardless of psychological issues about animals’ organ transplantation, the recipient prefers other people to be unaware of transplant as much as possible. So respecting confidentiality in transgenic animal’s organ transplant to human is very important. Nonetheless, considering that the recipient is infected with infections or dangerous diseases, it seems to be impossible to keep such a issue secret, therefore, one can regard this case as an exception in confidentiality due to endangering not only the welfare but also life of all.

### Collective human-based criticism

As said before, the range of possible side-effects of this process is not just limited to the recipient, and it also has impact on the relatives and the society. About recipient’s relatives, there is the possibility of transmission of virus or disease-causing factors, especially to the spouse or the sexual partner. Consequently, they should also be informed about the process of transplant and its side effects. Here, a new form of the informed consent is put forward, in which the addressee is asked not only the patient, but also some people who are in the close contact with the recipient (8).

In the case of some infectious diseases occur and spread in the society, especially in epidemics, many people will be involved. Therefore, some critics consider transgenic animals’ organ transplant to human against the health-care policy which aims to keep majority of society members healthy. In this way, the benefits of the recipient patient conflicts with the society. Although there is yet no exact limit to the person can endangers others’ benefits to achieve his/her own (23).

Furthermore, if transgenic animals’ organ transplantation into human is accepted, body organs will be considered like properties for selling and purchasing. Along with this, altruistic donation will gradually disappear from the realm of values.

### Animal-based ethics

Though in the history of philosophy, due to the dominance of human-centered schools of thought, animals have not been directly considered as the subject of ethical considerations, but with the help of ideas of Jeremy Bentham, the founder of Utilitarianism School, animals were introduced as residents of ethical society. Contrary to what Kant have thought about rationality as the only factor to be a member of moral community, Bentham have believed that sentiency, the ability to feel pain and pleasure, or positive or negative experiences, is enough for having a moral status. Since the animals are sentient, they can be counted as subject of ethics (26). After him, many philosophers have divided the moral status of animals in to two groups. Follower of *animal rights* considers them as holders of rights and they should be considered the same issues as the similar interests of human. On the other hand, the proponents of the *animal welfare* believed that human has a right to use animals in order to meet his needs if the animal suffering and the costs of use is less than the benefits to humans. The latter school is more accepted and welcomed in many aspects. The animal welfare can be summarized in three "R"s: *reduction, replacement,* and *refinement*, which are accepted as principles of using lab animals for the research. Regarding the use of animals’ organs in transplantation into human, some of the supporters of the *animal rights* like Tom Regan have disagreed strongly about human’s using animals for any kind of their needs through emphasizing on equal rights of animal and human being. As a result, from their point of view, torturing and killing animals to transplant their organs to human beings is unethical and means lowering animals’ status to things or to a tool box (27).

Along with this point of view, another group of animal ethics philosophers, especially utilitarian philosophers like Peter Singer, have not refused the idea of using animals for saving human life, and in contrast, only through attribution requirements, they have tried to limit and to decrease the quantity and quality of such a use. From their viewpoint, according to justified need or necessity, and if the benefits overcomes the degree of pain and suffering to animals, they can be used to meet human’s needs while treating them properly (28).

However, this group believes that transgenic animals’ organ transplantation into human is also criticized because it is against the concept of animal welfare (29).

Regarding this concept, two definitions have been given. In the broad definition, animals should be kept safe from some negative factors like thirst, hunger, pain, suffering, and psychological tensions. In transgenic animals’ organ transplantation into human, due to series of experiments, much pain and suffering are imposed on these animals during various steps of this technique. In addition, since there is the possibility of virus transference from animal to human, during their life-time, these animals should be raised with special diets and in supervised environments specified for their growths (30).

Based on the broad definition, the goal, in addition to keeping the animal away from negative factors, is to give a chance to animal to show its species-specific behavior and live according to its type. In other words, in this definition of animal welfare, the concept of being natural is highly emphasized (31). Therefore, genetic modification of animals is basically the total destruction of their welfare because the integrity of animal genome is manipulated to create an animal which is different from its natural type.

Furthermore, in order to prevent transfer of any kind of virus, these animals should be kept in a completely quarantined place and be imprisoned. This has caused a lot of ethical criticism, because keeping animals imprisoned in a place that is not similar to its natural habitat, far from any interaction with its kind, takes its chance to live naturally.

The broad definition of animal welfare is close to the animal right school, so, it seems that welfare of the animals cannot be provided with this definition in most of the farms and labs. For example keeping cows in closed areas, milking them with new machineries and feeding them with concentrated synthetic foods are totally far from their nature, but are practiced in almost all farms and ranching facilities. Same thing happens in all the labs which work with lab animals. In almost all of the animals at homes, lab animals are kept in small cages and are feed with synthetic food. Water is available for them through a special tool, and the humidity, light and temperature are leveled. So, there seems to be same problems against animal welfare in broad definition in all the animal labs and nothing is special for genetically manipulated animals.

Another issue is the conflict between transgenic animals’ organ transplantation into human and the triple R principles over working on lab animals. These principles emphasizes on using substitute methods and the obligation to use the fewest possible number of animals in lab in best welfare conditions (31). Consequently, organ transplantation from transgenic animals not only does not reduce the animal use but causes that more animals are kept in conditions contradicting with the concept of welfare. But it has to be said that the triple R refers to as much as possible. For example reduction means if the research can be done with 9 animals, 10 animals must not be used. With this manner, producing and using the transgenic animals as far as needed is justified.

## Conclusion

Considering what is said, although, transgenic animals’ organ transplantation into human can be scientifically considered so hopeful regarding the ethical issues, it is not verified at present. Regardless of general criticism, which can be attributed to many biological-technical dimensions, this treatment method raises many questions from both human-based and animalbased ethical points of view.

In the human-based side, it seems that from the viewpoint of precautionary principle and common good, it is better to keep this issue in the experimental level and clinical trials in a limited range due to lack of scientific certainties including diseases manifestations, virus transference from animal to human, its transference or spread in the society and also the degree of pain which is imposed on the patient and his/ her relatives. Following the steps of trials, with increasing human’s knowledge and by finding solutions for existing problems on the way to transplant and also a deeper acceptance of this issue in the society, its side-effects are reduced as much as possible.

From the animal-based side, the supporters of animal welfare school believe that starting this treatment process will destroy animal welfare from broad dimensions. This happens because its genetic structure is modified and therefore, the animal will not have the chance to live its instinct and will be deprived of the least factors necessary to live like being safe from pain and suffering. Therefore, even from the viewpoint of these philosophers who believe in the use of animals when necessary, this method at its present level is neither justified nor verified from the ethical point of view, but if its benefit and safety is proved, compromising the animal welfare to save a human life is justifiable.

Consequently, considering the above mentioned facts, we can conclude that within the current situation, transgenic animals’ organ transplantation into to human is not ethically justified; therefore, it needs to develop further .
